# Monitoring People With COVID-19 at Home With the COVIDFree@Home Program: Feasibility Cohort Study

**DOI:** 10.2196/69140

**Published:** 2025-10-08

**Authors:** Andrea S Gershon, Alex Mariakakis, Eyal de Lara, Joseph Munn, Maryann Calligan, Daniyal Liaqat, Salaar Liaqat, Junlin Chen, Teresa To, Philip W Lam, Andrew Simor, Adrienne K Chan, Nisha Andany, Sameer Masood, Nick Daneman, Tiffany Chan, Christopher Graham, Vikram Comondore, Andre de Moulliac, Alice Y Tu, Robert Wu

**Affiliations:** 1 Department of Medicine, Temerty Faculty of Medicine, University of Toronto, Toronto, ON, Canada; 2 Department of Medicine, Sunnybrook Health Sciences Centre, V1 06, 2075 Bayview Avenue, Toronto, ON, M4N 3M5, Canada, 1 4164804758, 1 4164805153; 3 Dalla Lana School of Public Health, University of Toronto, Toronto, ON, Canada; 4 ICES, Toronto, ON, Canada; 5 Department of Computer Science, University of Toronto, Toronto, ON, Canada; 6 Medicine, University Health Network, Toronto, ON, Canada; 7 Vector Institute for Artificial Intelligence, Toronto, ON, Canada; 8 Child Health Evaluative Sciences, The Hospital for Sick Children, Toronto, ON, Canada; 9 Department of Medicine, Trillium Health Partners, Toronto, ON, Canada; 10 Department of Medicine, William Osler Health System, Brampton, ON, Canada; 11 Department of Medicine, Toronto Metropolitan University, Toronto, ON, Canada; 12 Division of Respirology, McMaster University, Hamilton, ON, Canada; 13 IQVIA (United States), Durham, NC, United States

**Keywords:** COVID-19, remote monitoring, mobile application, oxygen saturation, pandemic

## Abstract

**Background:**

During the COVID-19 pandemic, many with acute infection isolated at home, with a small but significant number requiring hospitalization. At the time, since the pathogen was fairly unknown, clinicians were uncertain about which patients would rapidly deteriorate and need hospitalization. We developed the COVIDFree@Home smartphone app and clinician dashboard to monitor and support people managing at home with acute COVID-19 infection. It was uncertain whether such an app would be used by patients and whether it would support patient care. This knowledge would inform telemedicine and digital health tools being used to deliver care to patients remotely at that time.

**Objective:**

This study aimed to determine the feasibility of using a smartphone app and clinician dashboard for remote clinical monitoring of people with COVID-19 at home.

**Methods:**

A feasibility study set at 3 hospital sites (University Health Network, Sunnybrook Health Sciences Centre, and Trillium Health Partners) between 2020 and 2022 was conducted. Participants newly diagnosed with COVID-19 were asked to enter data into a smartphone app called COVIDFree@Home twice daily for 10 days while isolating at home. Their data, including symptoms, temperature, and oxygen saturation, were monitored on a clinician-facing dashboard. The primary outcome of feasibility was the number of patients who used the app. We also examined patient satisfaction through a survey questionnaire.

**Results:**

A total of 431 patients were recruited, out of which 229 (56.5%) were females and the average age was 38.9 (SD 12.8) years. There were 376 (87.2%) participants who used the app to report symptoms or oxygen saturation at least once. Among these participants, 373 (99.2%) reported symptoms and 363 (96.5%) reported oxygen saturation. Participants reported symptoms an average of 1.7 (SD 1.1) times per day for a median of 5 (IQR 3‐8) days. Oxygen saturation levels were reported 1.5 times per day for a median of 6 (IQR 4‐9) days. There were 19 hospitalizations (4.4%) among study participants. Most patients felt comfortable using the app, felt reassured their data was being monitored and did not have privacy concerns.

**Conclusions:**

Patients with acute COVID-19 infection engaged with a remote home monitoring platform, however, not at the recommended frequency or duration. Remote patient monitoring of acute respiratory infection appears viable and can offer patients reassurance. It has the potential to reduce strain on the health care system during future pandemics, but further evidence is required to demonstrate improved health outcomes.

## Introduction

The COVID-19 pandemic had an unprecedented global impact on human health and health care systems. The influx of patients overburdened hospitals and led to disruptions in health care, demanding quick adaptations in how care was delivered [1,2]. Particularly in the initial phase of the pandemic, people with COVID-19 isolated at home, with a small but significant number deteriorating and requiring hospitalization. With home isolation becoming common and health care resources being limited, people turned to remote care solutions to support patients outside of hospital settings [3]. Since the pathogen was fairly unknown, clinicians were uncertain about which patients would rapidly deteriorate and need hospitalization. Many clinicians were concerned about a phenomenon referred to as “silent hypoxia,” wherein patients had low oxygen saturation levels without experiencing shortness of breath. This led to groups recommending that patients be followed at home with oximeters [4-6].

In response to this, a transdisciplinary team of researchers, computer scientists, and clinicians (infectious disease, respirology, and general internal medicine) developed the COVIDFree@Home remote monitoring program to support and study people recovering from COVID-19 for the duration of their illness. This program consisted of a patient-facing smartphone app for data collection and a clinician-facing dashboard that enabled health care providers to remotely monitor a roster of patients. The uptake of such a program by patients, as well as their satisfaction with these technologies in the context of the pandemic, was unknown.

During the pandemic, telemedicine and digital health tools subsequently emerged as means to deliver care to patients [7], but few were evaluated. A systematic review of remote monitoring programs for COVID-19 found 23 descriptive reports of remote monitoring systems that varied significantly in the patient groups they studied, the technology they used, and the outcome monitored [8]. Of the 23 reports, only 9 assessed patient satisfaction, and only 12 assessed patient adherence. None evaluated effectiveness. Another review examined 241 studies of remote monitoring of patients with COVID-19 and found a lack of evidence of effectiveness [9]. Of these, only 73 evaluated patient experience. This review did not assess adherence. Lack of evaluation of previous remote monitoring programs represents a lost opportunity to learn from past experiences to improve monitoring in the future.

We conducted a prospective study to document the feasibility of using the COVIDFree@Home program for patients with acute respiratory infection, specifically those newly diagnosed with COVID-19 infection isolating at home. Our secondary objective was to determine patient satisfaction with the monitoring. This information can be used to inform future remote clinical monitoring solutions.

## Methods

### Study Design

We conducted a multisite, prospective clinical study to evaluate the feasibility of using a smartphone app for remote clinical monitoring of patients with COVID-19 recovering at home. Patients were recruited from 3 Ontario health networks: University Health Network, Sunnybrook Health Sciences Centre, and Trillium Health Partners from October 12, 2020, to June 23, 2022 (ClinicalTrials.gov NCT04453774).

### Participants

Patients were recruited from COVID-19 ambulatory testing sites at the 3 hospital networks. In the course of regular clinical care, those who received a positive SARS-CoV-2 polymerase chain reaction test result were contacted by phone by clinicians for a routine online synchronous medical assessment. Patients were invited to participate in our study during these online assessments.

For those with acute COVID-19 who were interested in participating, a member of the research team contacted them by phone to go through the inclusion and exclusion criteria and informed consent process. Patients were invited to participate if they were 18 years of age or older, English-speaking, within 6 days of testing positive for COVID-19, and experiencing symptoms of COVID-19. Those who were receiving palliative care or had significant comorbidities that would confound symptoms were excluded but still received regular care. Patients were followed for the duration of their isolation period (10 days after symptom onset) or until their symptoms resolved, whichever was earlier.

### Study Intervention: COVIDFree@Home Program

The COVIDFree@Home program consisted of a patient-facing smartphone app and a clinician-facing dashboard. Patients were asked to download the smartphone app to their personal devices; if they did not own one, smartphones were available to be loaned by the study team, but this did not occur in our study. Patients were also provided with a thermometer and digital pulse oximeter.

**Figure 1.**
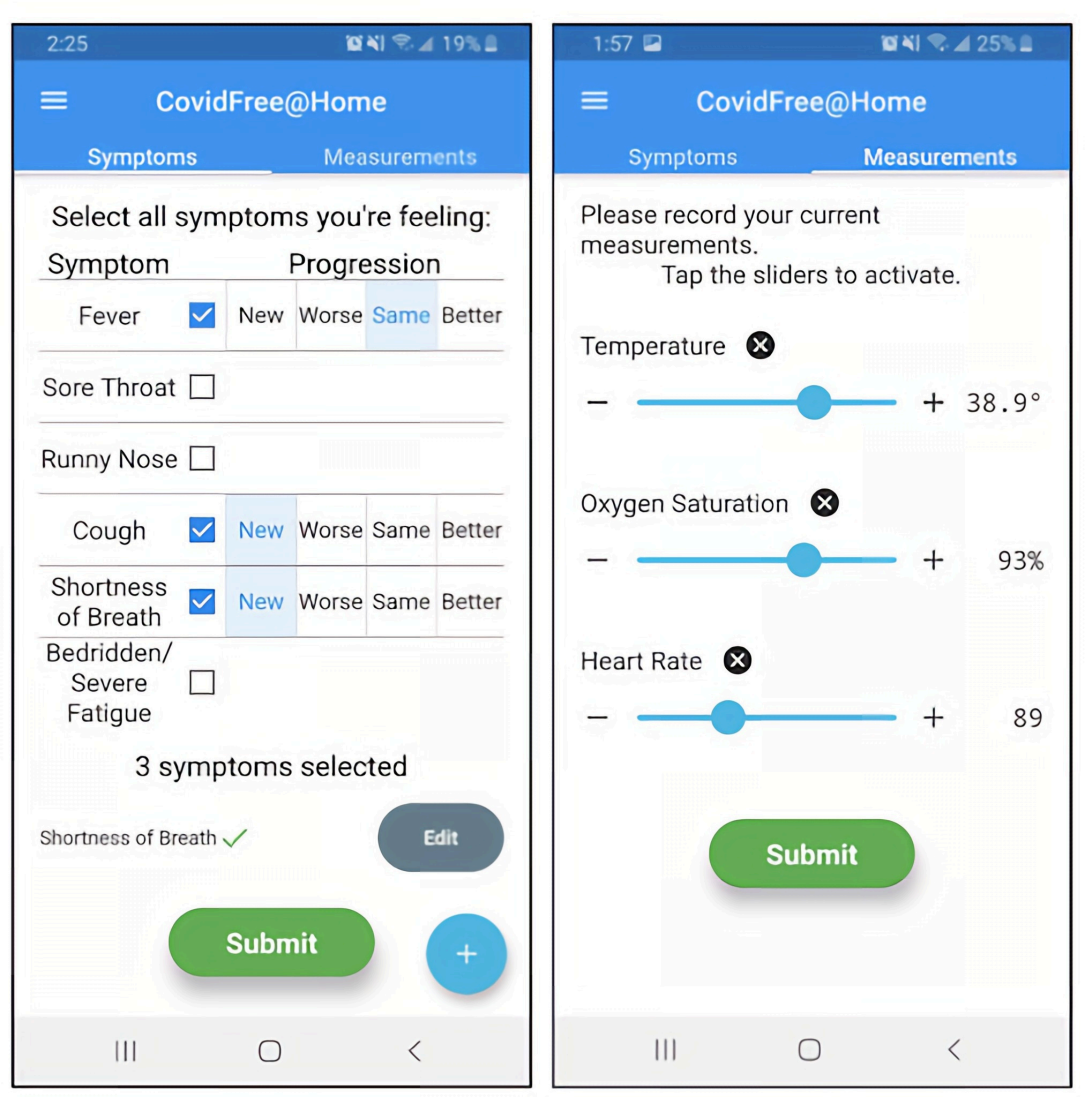
Screenshots from the patient-facing COVIDFree@Home mobile app, which allows patients to report their symptoms, temperature, oxygen saturation, and heart rate.

After taking readings with the devices, they were instructed to input the resulting oxygen saturation level, heart rate, and temperature data into the app while also noting the symptoms they were experiencing. Patients were asked to do this twice daily for routine assessments and input additional records whenever they experienced a notable change in health (Figure 1). Patients were instructed that their information was reviewed daily, that they should not depend on it for urgent medical attention, and that they should use their usual processes (eg, calling their family physician or going to the emergency department) as appropriate.

**Figure 2.**
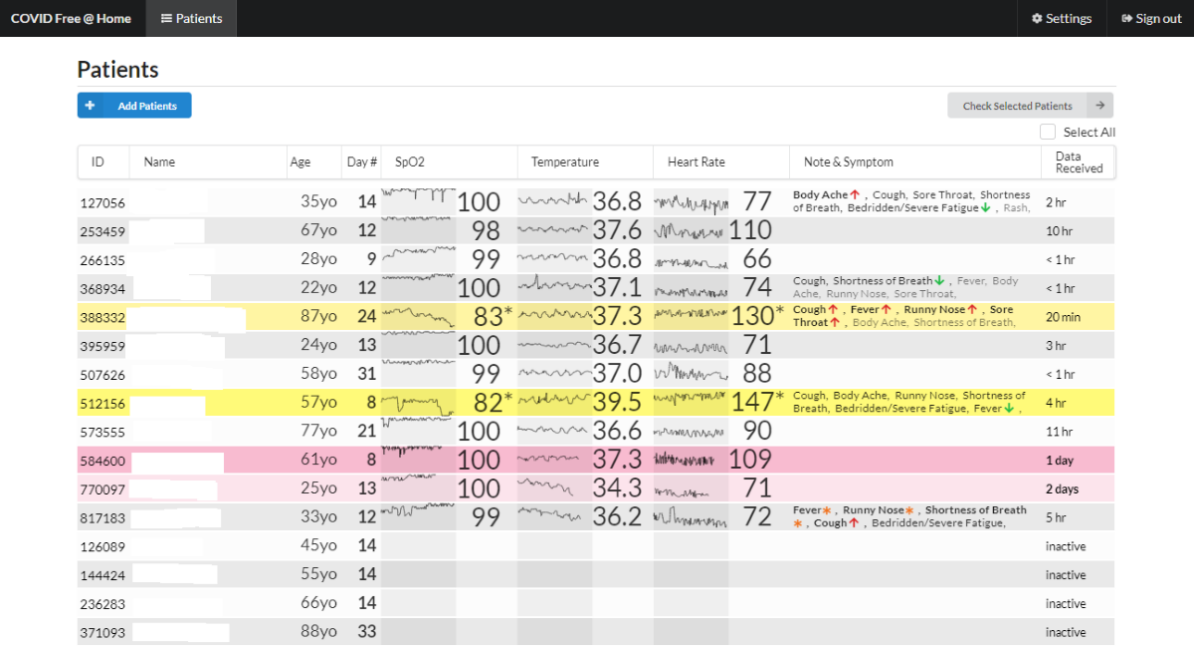
Screenshot of a page from the clinician-facing COVIDFree@Home dashboard. This screen displays age, oxygen saturation, temperature, heart rate, and notes on symptoms for a roster of patients being monitored.

Individual patient information was displayed on a clinician-facing dashboard accessed via a secure website by clinicians (Figure 2). Out-of-range values were visually highlighted to enable rapid identification of patients who were potentially deteriorating. Email alerts were also sent to clinicians automatically for out-of-range values as notifications. In addition, physicians reviewed their assigned patients’ data and then signed off on it at least once per day. This sign-off could be seen by patients on their app, thus providing reassurance that their data was being monitored. To further facilitate interaction between patients and clinicians, there was a messaging feature between the app (patients) and the dashboard (clinicians). A physician monitored patients at each site. Escalation procedures were at the discretion of the monitoring physician but usually included calling the participant with a potential request that they go to the emergency department, asking them to keep monitoring, or providing reassurance that their health status is normal.

### Study Outcomes

As this was a feasibility study, the primary outcome was adherence to using the app at least once to report symptoms or oxygen saturation. Reporting of oxygen saturation was also selected as the main outcome as it was the most relevant vital indicator for our targeted population. Secondary outcomes included the number of days the app was used and the average entries per day. Patients were asked to report if they were hospitalized through the app. We also asked patients to report on their experience using the smartphone app in an end-of-study questionnaire. In this questionnaire, they were asked to rate their agreement with a series of statements about using the COVIDFree@Home app on a 5-point Likert scale ranging from 1=strongly disagree to 5=strongly agree. Participants were also given the opportunity to provide free-form feedback in a text field.

### Patient Characteristics

Demographics and comorbidities were collected via questionnaires during study enrollment. Ethnicity was collected to understand the differences among patients with COVID-19 who might experience marginalization.

### Data Analysis

We used descriptive statistics to explore the characteristics of our patient population, usage of the app, and other secondary outcomes including patient experience. Quantitative data from the questionnaire were summarized according to median and IQR using Python (Python Software Foundation). Missing data and those lost to follow-up were reported. Free-form text data was reviewed using thematic coding, and representative quotes were selected for each theme.

### Ethical Considerations

This study received Research Ethics Board approval through Clinical Trials Ontario (study ID 3185) and the associated study sites. Written consent was obtained from all patients prior to participation. Privacy and confidentiality protection were in place with study data deidentified. There was no compensation for participation.

## Results

### Patient Population

We consented 431 patients to participate in the study and provided them with the COVIDFree@Home app. Of these, 229 (56.5%) were reported as female, and the average age was 38.9 (SD 12.8) years (Table 1). A total of 185 of the 431 (42.9%) patients reported at least 1 comorbidity, with the most common being obesity (14.9%), asthma (8.6%), hypertension (7.1%), and diabetes (4%). Of the 98 participants who reported their ethnicity (22.7%), 30 (30.6%) identified as white and 39 (39.8%) as Asian.

**Table 1.** Baseline characteristics of the COVIDFree@Home project participants recruited at 3 sites in Ontario, Canada, between October 12, 2020, and June 23, 2022.

Characteristics	Using the app (n=376)	Not using the app (n=55)	Total (n=431)
Sex, n (%)			
Female	194 (51.6)	35 (63.6)	229 (56.5)
Missing or not reported^a^	—^b^	—	26
Age (years), mean (SD)	38.6 (12.5)	40.8 (14.5)	38.9 (12.8)
Missing or not reported	—	—	18
Self-reported comorbidities, n (%)			
Obesity	53 (15.3)	6 (12.2)	59 (14.9)
Asthma	31 (8.9)	3 (6.1)	34 (8.6)
Hypertension	26 (7.5)	2 (4.1)	28 (7.1)
Diabetes	14 (4)	2 (4.1)	16 (4)
Mental illness	14 (4)	4 (8.2)	12 (3)
Missing or not reported	—	—	35
Self-reported ethnicity, n (%)			
Asian	—	—	39 (39.8)
White	—	—	30 (30.6)
Middle Eastern	—	—	17 (17.3)
Black	—	—	6 (6.1)
Latin American	—	—	3 (3.1)
Other	—	—	3 (3.1)
Missing or not reported	—	—	333
COVID-19 variant by date^c^, n (%)			
Alpha or Beta	209 (55.6)	34 (61.8)	243 (56.4)
Delta	55 (14.6)	9 (16.4)	64 (14.8)
Omicron	112 (29.8)	12 (21.8)	124 (28.8)
MRC^d^ Dyspnea scale at baseline, n (%)			
No dyspnea	279 (86.9)	41 (87.2)	320 (87)
Slight dyspnea	34 (10.6)	2 (4.3)	36 (9.8)
Moderate dyspnea	8 (2.5)	3 (6.4)	11 (3)
Severe dyspnea	0 (0)	1 (2.1)	1 (0.3)
Missing or not reported	—	—	63

a Not reported due to large number of missing data.

b Not available.

c Determined by most prevalent variant at date of enrollment.

d MRC: Medical Research Council.

### App Use

**Figure 3.**
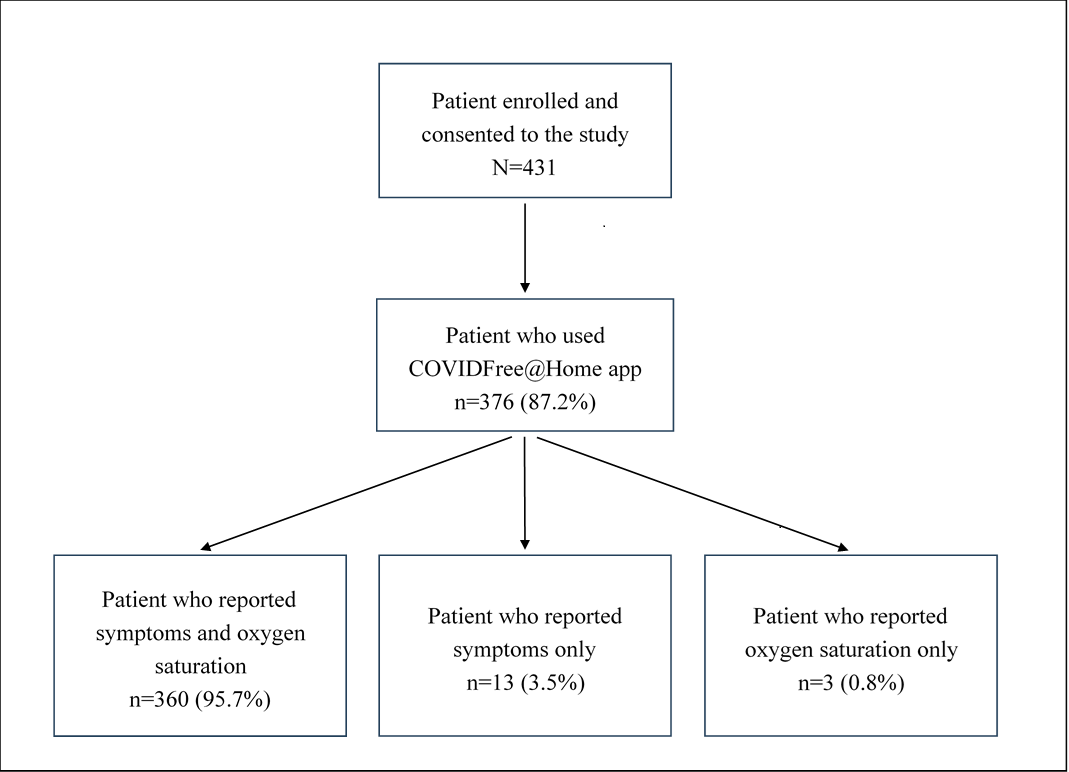
Summary of participant engagement with various parts of the COVIDFree@Home app.

Overall, there were a total of 4121 entries for symptoms (average per patient 11, SD 12.9), 4128 for oximetry and heart rate (average per patient 11.4, SD 12.5), and 3598 for temperature (average per patient 10.3, SD 12.3). The average time that passed before participants began using the app after receiving a positive polymerase chain reaction test result was 2.1 (SD 3) days, including the time participants were invited and enrolled into the study. There were 376 participants (87.2%) who used the app at least once (Figure 3; Table 2). Among these participants, 373 (99.2%) reported symptoms, and 363 (96.5%) reported oxygen saturation. On average, participants entered symptoms 1.7 (SD 1.1) times and measured oxygen saturation 1.5 times (SD 1) per day. Most participants (346/376, 92%) reported symptoms and oxygen saturation for at least 6 days after enrollment.

**Table 2.** App usage of patients who used the app at least once (n=376).

Usage **measure**		**Values** (n=376)
Participants who used the app to report symptoms, n (%)		373 (99.2)
Daily measures taken with the app, mean (SD)		1.7 (1.1)
Total days of app use, median (IQR)		5 (3-8)
Days from positive test to enrollment, mean (SD)		2.1 (3)
Days from enrollment to first entry, mean (SD)		5.5 (2.4)
Participants who used the pulse oximeter, n (%)		363 (96.5)
Daily measures taken using the pulse oximeter, mean (SD)		1.5 (1)
Total days of oximeter use, median (IQR)		6 (4-9)

Of the 363 participants who recorded oxygen saturation, 45 (12.4%) experienced at least 1 oxygen saturation level below 92%, and 13 (3.6%) below 88%. Among all study participants, 19 of 431 (4.4%) were subsequently hospitalized, and no deaths were reported.

### Patient Experience

Of the 376 participants who used the COVIDFree@Home app, 143 (38%) completed the patient experience questionnaire at the end of the study (Table 3). On a 5-point Likert scale ranging from “1: strongly disagree” to “5: strongly agree,” participants were comfortable using the app (Q2: median 4, IQR 3‐5), did not need assistance (Q4: median 1, IQR 1‐1), and felt reassured their data was being monitored (Q7: median 5, IQR 4‐5). Most did not have privacy concerns about reporting their health information through the app (Q12: median 1, IQR 1‐2). Eighty-one respondents (56.6%) agreed that the app helped manage their COVID-19 infection, and 84 (58.7%) agreed that it improved communication with their health care providers. Participant free-form feedback was generally positive, with many noting the reassurance that monitoring provided (Textbox 1). Suggestions by some participants included being able to view one’s own data, particularly with trend lines to show historical changes.

**Table 3.** Questionnaire that participants completed to report their experience with the COVIDFree@Home app. Each question was answered along a 5-point Likert scale (1=strongly disagree, 2=somewhat disagree, 3=neither disagree nor agree, 4=somewhat agree, 5=strongly agree).

Statements	Median **response** (**IQR**)
I have used a virtual app for my medical care before using the COVIDFree@Home app	1 (1-3)
I was comfortable using electronic apps for my health prior to participating in this study	4 (3-5)
After participating in this study, I am comfortable using electronic apps for my health	5 (4-5)
I needed a lot of help to use the app (from friends, family, technical support)	1 (1-1)
I had technical difficulties due to technical problems with the app	1 (1-2)
I had difficulties due to internet connectivity	1 (1-1)
I felt reassured knowing my health data was being monitored	5 (4-5)
Entering data twice a day is a burden	2 (1-3)
The app helped me manage my COVID infection	4 (3-5)
The app made me more aware of my symptoms	4 (4-5)
The app improved communication with my health care provider	4 (3-5)
I had privacy concerns that my health information was being uploaded	1 (1-2)
I would like to be able to see the information that I entered in the app	4 (3-5)

Textbox 1. **Selected feedback from participants on the COVIDFree@Home app.**

- “It gave me an unbelievable amount of piece (sic) of mind to see that someone was watching my entries. My biggest fear was not knowing if I should seek further medical attention.”
- “This app was very important to my family. My wife had much worse symptoms than I did and the feedback we received gave us peace of mind that we were on the road to recovery.”
- “I was very impressed with the response from the medical team when my symptoms became a concern. Using this app kept me out of the hospital and provided me with oxygen and puffers to help with my recovery.”
- “It would have been powerful (and helpful to me) to see a trend analysis to show me how my data was tracking over time. Not sure why you wouldn't empower patients to see their own data and how it changes. Brings much better awareness to the situation.”

## Discussion

### Principal Findings

We conducted a multisite, prospective clinical study to determine the feasibility of using a smartphone app to remotely monitor patients with respiratory disease isolating at home during a pandemic. We found that 87% of participants reported symptoms and oxygen saturation using the app at least once and continued to use it for a median of 5 days. In terms of user experience, the feedback received from participants was positive overall. In assessing patient satisfaction with app-based monitoring, we found that most respondents were comfortable using the platform, found it helpful in managing their COVID-19 condition, and reported minimal privacy concerns. Taken together, these findings highlight the feasibility of using a smartphone app to support patients in managing their health and offering emotional comfort through remote monitoring during a pandemic.

The burden of COVID-19 infection has been dramatically reduced due to less aggressive variants, vaccine development, and new therapeutics for COVID-19. Unlike early in the pandemic, COVID-19 infection is now more predictable, and hospitalization is less likely. However, 6 million people are still hospitalized and between 400,000 to 700,000 people die from COVID-19, influenza, and respiratory syncytial virus per year globally [10,11]. Most of these are frail, immunosuppressed, or both. We need remote monitoring that can help these people and reduce the burden on health care systems. Our findings can be used to create and improve upon monitoring systems that patients will use and that are effective.

Our findings that most participants used an app to report health information and were satisfied with the experience are consistent with the literature. In a systematic review of 23 studies using remote patient monitoring in people with acute COVID-19, adherence was generally high in the 12 that evaluated it [8]. Similarly, patient acceptance was reported in the 9 studies that evaluated it. In a large review of 164 telemonitoring papers, 73 mentioned patient experience and found it positive [9]. Of note, this review found no evidence of effectiveness in improving outcomes of reduced ED visits or hospitalizations. While a systematic review of reviews of mobile apps for COVID-19 found many reviews of many apps, few focused on remote monitoring of infection [12]. A recent review of remote monitoring of COVID-19 found that 4 of 13 total studies used a smartphone self-monitoring app [13-17]. Of these, 1 reported satisfaction which was predominantly positive [14], and 1 provided information on usage [15]. Our study adds value by providing a prospective analysis of the feasibility, usage, and patient experience of remote monitoring using a smartphone app—a format of remote monitoring for which studies of feasibility have been limited [18].

The strengths of our study were its ability to prospectively evaluate a real-world clinically active remote monitoring program. It also has limitations that warrant emphasis. First, we were not able to recruit all people who were seen with COVID-19 infection at our sites, and thus, our findings may not be generalizable to the general population. This was due to a combination of participants not being able or interested in participating. We found this to be worse during times when COVID-19 surged, and it was made worse because participants had to be enrolled in a limited window of time right after their infection was diagnosed. A second limitation was that—due to the nature of how our study was set up to accommodate the pandemic—we were unable to determine which patients did not engage with COVIDFree@Home. We also found low questionnaire response rates to some questions, namely participants’ ethnicity, limited our ability to collect complete demographic data. This left us unable to be certain who found our remote monitoring program inaccessible or unacceptable. For example, while older people are more likely to experience severe illness, we hypothesize that they may have been less likely to participate because they were less comfortable with technology due to diminished digital health literacy. A third limitation is that we were unable to determine the effectiveness of the intervention due to there being too few adverse clinical outcomes and a lack of control arm. Future studies should assess feasibility and effectiveness of remote monitoring in diverse socioeconomic groups including older populations. Fourth, we did not evaluate health care provider experience alongside patient experience. To scale such a system, it would be important to engage health care workers who would do the monitoring.

### Conclusions

Our multisite clinical study indicated that our remote patient monitoring intervention, the COVIDFree@Home app and clinician dashboard, was feasible and well-received according to quantitative usage metrics and qualitative feedback. Future research should use remote monitoring data to develop digital biomarkers that predict patient deterioration so that interventions can be implemented proactively to prevent hospitalizations [19].

## References

[1] Okafor NM, Thompson I, Venkat V (2025). Evaluating the feasibility, adoption, cost-effectiveness, and sustainability of telemedicine interventions in managing COVID-19 within low-and-middle-income countries (LMICs): a systematic review. PLOS Digit Health.

[2] Garfan S, Alamoodi AH, Zaidan BB (2021). Telehealth utilization during the Covid-19 pandemic: a systematic review. Comput Biol Med.

[3] Chaiyachati KH, Shea JA, Ward M (2023). Patient and clinician perspectives of a remote monitoring program for COVID-19 and lessons for future programs. BMC Health Serv Res.

[4] Teo J (2020). Early detection of silent hypoxia in Covid-19 pneumonia using smartphone pulse oximetry. J Med Syst.

[5] Lam PW, Sehgal P, Andany N (2020). A virtual care program for outpatients diagnosed with COVID-19: a feasibility study. CMAJ Open.

[6] Levitan RM, Kline JA (2020). Pulse oximetry as a biomarker for early identification and hospitalization of COVID-19 pneumonia. Acad Emerg Med.

[7] Shaver J (2022). The state of telehealth before and after the COVID-19 pandemic. Prim Care.

[8] Joyce D, De Brún A, Symmons SM, Fox R, McAuliffe E (2023). Remote patient monitoring for COVID-19 patients: comparisons and framework for reporting. BMC Health Serv Res.

[9] Cornelis J, Christiaens W, de Meester C, Mistiaen P (2024). Remote patient monitoring at home in patients with COVID-19: narrative review. JMIR Nurs.

[10] Mazur NI, Terstappen J, Baral R (2023). Respiratory syncytial virus prevention within reach: the vaccine and monoclonal antibody landscape. Lancet Infect Dis.

[11] Influenza (seasonal). World Health Organization.

[12] Holl F, Schobel J, Swoboda WJ (2024). Mobile apps for COVID-19: a systematic review of reviews. Healthcare (Basel).

[13] Lara B, Kottler J, Olsen A, Best A, Conkright J, Larimer K (2022). Home monitoring programs for patients testing positive for SARS-CoV-2: an integrative literature review. Appl Clin Inform.

[14] Annis T, Pleasants S, Hultman G (2020). Rapid implementation of a COVID-19 remote patient monitoring program. J Am Med Inform Assoc.

[15] Gordon WJ, Henderson D, DeSharone A (2020). Remote patient monitoring program for hospital discharged COVID-19 patients. Appl Clin Inform.

[16] Medina M, Babiuch C, Card M (2020). Home monitoring for COVID-19. Cleve Clin J Med.

[17] Yordanov Y, Dechartres A, Lescure X (2020). Covidom, a telesurveillance solution for home monitoring patients with COVID-19. J Med Internet Res.

[18] Pandit JA, Radin JM, Quer G, Topol EJ (2022). Smartphone apps in the COVID-19 pandemic. Nat Biotechnol.

[19] Sim I (2019). Mobile devices and health. N Engl J Med.

